# Effects of tumor-infiltrating lymphocytes on nonresponse rate of neoadjuvant chemotherapy in patients with invasive breast cancer

**DOI:** 10.1038/s41598-023-36517-2

**Published:** 2023-06-07

**Authors:** Xiao-Long Qian, Xiao-Qing Xia, Ya-Qing Li, Yu-Mian Jia, Yuan-Yuan Sun, Yuan-Ming Song, Hui-Qin Xue, Yan-Fei Hao, Jin Wang, Xiao-Zi Wang, Chen-Ying Liu, Xin-Min Zhang, Li-Na Zhang, Xiao-Jing Guo

**Affiliations:** 1 Department of Breast Pathology and Lab, Tianjin Medical University Cancer Institute and Hospital, National Clinical Research Center of Cancer, Key Laboratory of Cancer Prevention and Therapy, Tianjin’s Clinical Research Center for Cancer, Key Laboratory of Breast Cancer Prevention and Therapy, Tianjin Medical University, Ministry of Education, National Human Genetic Resource Sharing Service Platform, West Huanhu Road, Tianjin, 300060 China; 2 Department of Pathology, Cooper Medical School of Rowan University, Camden, NJ USA; 3 The Second Surgical Department of Breast Cancer, Tianjin Medical University Cancer Institute and Hospital, National Clinical Research Center of Cancer, Key Laboratory of Cancer Prevention and Therapy, Tianjin’s Clinical Research Center for Cancer, Key Laboratory of Breast Cancer Prevention and Therapy, Tianjin Medical University, Ministry of Education, National Human Genetic Resource Sharing Service Platform, Tianjin, 300060 China

**Keywords:** Cancer, Oncology, Risk factors

## Abstract

High level of tumor-infiltrating lymphocytes (TILs) can predict the rate of total pathological complete remission (tpCR) of breast cancer patients who receive neoadjuvant chemotherapy (NACT). This study focused on evaluating the data of patients whose primary tumor and/or lymph node metastasis show nonresponse (NR) to NACT, trying to provide a basis for the clinical decision which patients will develop NACT resistance. The study included breast cancers from 991 patients who received NACT. ROC curve analysis confirmed that TILs showed significant predictive value for NR of hormone receptor (HR)+HER2− and triple-negative breast cancer (TNBC). Among HR+HER2− breast cancer, TILs ≥ 10% was an independent predictor for low NR rate. Furthermore, positive correlation of TILs with Ki67 index and Miller-Payne grade, and negative correlation with ER and PR H-scores were only identified in this subgroup. In TNBC, TILs ≥ 17.5% was an independent predictor for low NR rate. The predictive value of low TILs on NR may facilitate to screen patients with HR+HER2− or TNBC who may not benefit from NACT. HR+HER2− breast cancer with low levels of TILs should be carefully treated with neoadjuvant chemotherapy, and other alternatives such as neoadjuvant endocrine therapy can be considered.

## Introduction

Neoadjuvant therapy (NAT) refers to systemic therapy before the implementation of local treatment such as surgery. Haagensen and Stout first proposed the concept of neoadjuvant chemotherapy (NACT), which was originally used as induction chemotherapy or initial chemotherapy for non-excisional, locally advanced breast cancer. The first goal of NAT for breast cancer patients is to reduce the tumor volume and stage, giving non-surgical breast cancer patients the opportunity to have surgical treatment^1,2^. The second target is to observe the sensitivity of tumor to drugs and guide follow-up adjuvant treatment^3^. The third purpose is to improve the prognosis of patients. To achieve pathological complete remission (pCR) can be used as a marker for NAT to improve the prognosis in breast cancer patients^3,4^.

Tumor-infiltrating lymphocytes (TILs) are leukocytes that leave blood stream and enter into tumor microenvironment. Researchers have carried out relevant studies in many cancer types to quantify these TILs and correlate their number with tumor characteristics and clinical consequences^5^. TILs are believed to represent local immune response, a key mechanism in controlling tumor growth and metastasis, and an independent prognostic indicator for many tumors^6^. However, its role is complicated, as scientific evidences indicate that subpopulation of TILs could help cancer cells to escape from the attack of host immune mechanisms. As a key representative of the immune interaction between host and tumor, TILs have been ideally placed for continued research into the determinants of immunogenicity, and response to immunotherapeutic approaches. Systematic evaluations on TILs may be able to guide the appropriate sequencing of therapies in breast cancer^7^.

Tumor response to NACT varies remarkably, from total pathologic complete remission (tpCR) to no response (NR)^8–11^. Here, we defined NR to NACT as cases without obvious chemotherapy response in the primary tumor or in metastatic lymph nodes. Although the association between NR and the prognosis of breast cancer by each molecular subtype is uncertain due to the lack of relevant research, cancer with NR is particularly problematic as it may reduce the chance of surgery by tumor progression.

Some studies have reported other possible negative effects of NACT in breast cancer. Karagiannis et al. found that the clinically validated prognostic markers for metastasis in breast cancer patients, the structures of the tumor microenvironment, increased under the effect of NACT, suggesting that residual tumor after NACT carries a higher risk of metastasis^12^. Keklikoglou et al.^13^ showed that in breast cancer models, chemotherapy elicits pro-metastatic extracellular vesicles which can enhance tumor metastatic capacity of breast cancer. These studies show that NACT has potential risky effect on patients while playing its therapeutic role. When tumor shows NR to chemotherapy, the risk is probably more prominent, as the remaining tumor volume is significantly large. If NACT for triple-negative breast cancer (TNBC) is not effective after 2–4 cycles of treatments, there is still much controversy over whether to choose a new neoadjuvant regimen^14^.

The magnitude of TILs is variable within and among subtypes of breast cancer^15^. Several studies have shown that in HER2+ breast cancer and TNBC, high TILs are associated with better prognosis^7,16–18^ and increased tpCR^19,20^. In hormone receptor(HR)+HER2− cancers, their association with the prognosis of patients with adjuvant therapy^21^ or NAT remains controversial^22^. To provide a basis for clinical decision making about NACT or other neoadjuvant modalities, this study analyzed the relationship between TILs and the tpCR rate, NR rate of NACT, the relationship between TILs and Miller–Payne grade (MP grade) reflecting the response of primary invasive tumor to chemotherapy, as well as the relationship between TILs and other conventional pathological indices of breast cancer.

## Results

### Baseline parameters and distribution of TILs in clinicopathological subgroups

**Figure 1:**
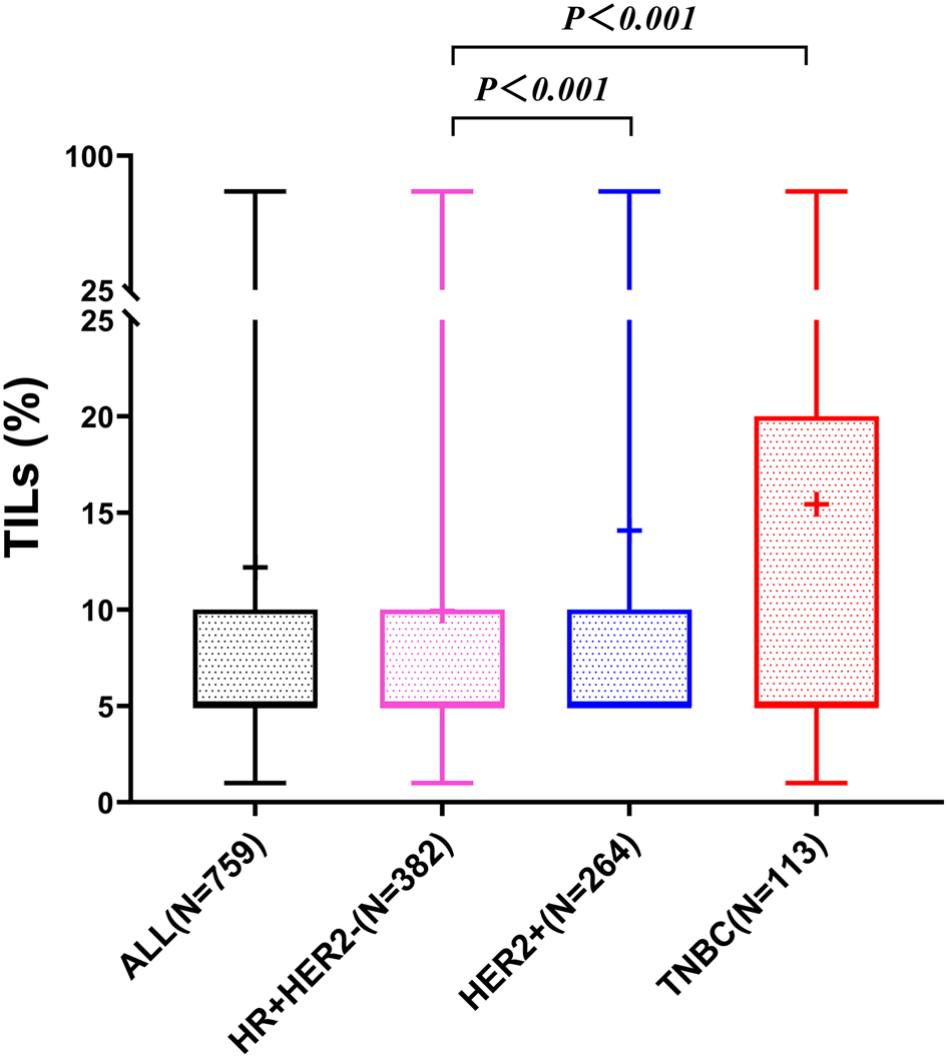
TILs of needle biopsy specimens in different molecular subtypes of breast cancer. *TILs* tumor-infiltrating lymphocytes.

The results of TILs’ evaluation in pre-neoadjuvant biopsy specimens were included in 759 of the 991 cases (Table 1). Using the cutoff point of TILs ≥ 10%, 501 (66.0%) tumors had low TILs and 258 (34%) had high TILs. High TILs was more frequently found in invasive ductal carcinoma (252/720; 35.0%) than in invasive lobular carcinoma (3/16; 18.8%; P = 0.0039). Similarly, high TILs was more common in TNBC (56/113; 49.6%) and HER2+ carcinomas (101/264; 38.3%) than in HR+HER2− cancer (101/382; 26.4%; p < 0.001). The same was observed in cancer with tpCR (50/93; 53.8%) group than that in the non-tpCR group (208/666; 31.2%; p < 0.001). High TILs was less frequent in the NR cancer group (20/105; 19.0%) than that in the non-NR cancer group (238/654; 36.4%; p < 0.001, Table 1). In comparing the raw TILs percentage (a continuous variable) among different molecular subtypes of cancer, it was found that TILs in TNBC and HER2+ breast cancer were significantly higher than that in HR+HER2− breast cancer (Z = 4.794; P < 0.001 and Z = 4.068; P < 0.001, respectively, Fig. 1).

**Table 1:** Baseline parameters and distribution of TILs in clinicopathological subgroups.

	Number of patients	TILs low (< 10%)	TILs high (≥ 10%)	*P* value*
Age (n = 759)				
≤ 35 years	72 (9.5%)	47 (65.3%)	25 (34.7%)	0.891
> 35 years	687 (90.5%)	454 (66.1%)	233 (33.9%)	
Tumor types (n = 759)				
Ductal	720 (94.9%)	468 (65.0%)	252 (35.0%)	0.039
Lobular	16 (2.1%)	13 (81.3%)	3 (18.8%)	
Others^a^	23 (3.0%)	20 (87.0%)	3 (13.0%)	
Molecular subtypes (n = 759)				
HR+HER2-	382 (50.3%)	281 (73.6%)	101 (26.4%)	< 0.001
HER2+	264 (34.8%)	163 (61.7%)	101 (38.3%)	
TNBC	113 (14.9%)	57 (50.4%)	56 (49.6%)	
Operation types (n = 759)				
Breast conserving	49 (6.5%)	27 (55.1%)	22 (44.9%)	0.096
Radical	710 (93.5%)	474 (66.8%)	236 (33.2%)	
tpCR (n = 759)^b^				
Yes	93 (12.3%)	43 (46.2%)	50 (53.8%)	< 0.001
No	666 (87.7%)	458 (68.8%)	208 (31.2%)	
NR (n = 759)^c^				
Yes	105 (13.8%)	85 (81.0%)	20 (19.0%)	< 0.001
No	654 (86.2%)	416 (63.6%)	238 (36.4%)	

*χ^2^ test.

^a^Includes all tumours that were neither ductal nor lobular.

^b^tpCR (total pathological complete remission) was defined as the absence of residual invasive cancer in breast and axillary nodes following neoadjuvant therapy. Specimens after neoadjuvant with remaining tumor cells in the vessels / lymph nodes are non pCR.

^c^NR (nonresponse to neoadjuvant chemotherapy) were defined as cases without obvious chemotherapy response in primary tumor (Miller-Payne Grade 1: No change or some alternation to individual malignant cells but no reduction in overall cellularity) or in metastatic lymph nodes (Classified as grade N–C according to Sataloff grading system for lymph nodes: lymph nodes with cancer cell metastasis while without chemotherapy response) or both.

### High TILs associated with higher tpCR rate and lower NR rate in overall NACT cases, regardless of molecular typing

Among the 991 cases receiving NACT, tpCR was achieved in 151 cases (15.2%), and NR remained in 131 cases (13.2%).

**Figure 2:**
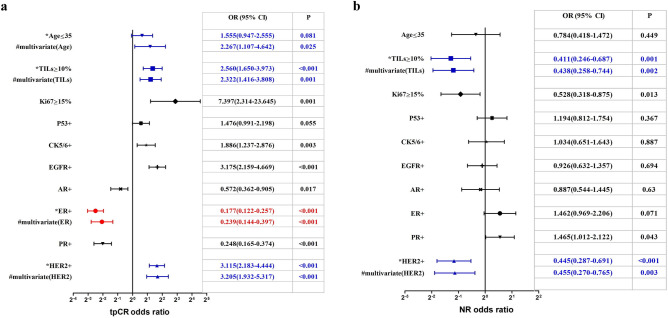
Analysis of factors on tpCR (**a**) and NR (**b**) rates in all breast cancer cases after NACT. *tpCR* total pathological complete remission, *NR* nonresponse to neoadjuvant chemotherapy, *NACT* neoadjuvant chemotherapy.

Univariate analysis of the tpCR rate of breast cancer cases after NACT was shown in Fig. 2a. High TILs were associated with higher tpCR rate {OR (95% CI): 2.560 (1.650–3.973); P < 0.001}. Multivariate analysis showed that high TILs {OR (95% CI): 2.322 (1.416–3.808); P = 0.001}, along with young age (≤ 35 years old) and HER2 positivity were independent predictor for higher tpCR rate in NACT group, while ER positivity was the only independent predictor for low tpCR rate (Fig. 2a).

Univariate analysis of the NR rate of breast cancer cases after NACT was shown in Fig. 2b. High TILs were associated with a lower NR rate {OR (95% CI): 0.411 (0.246–0.687); P = 0.001}. Multivariate analysis showed that high TILs {OR (95% CI): 0.438 (0.258–0.744); P = 0.002}, together with HER2 positivity were independent predictor for a lower NR rate in the NACT group (Fig. 2b).

### TILs associated with routine pathological index and high TILs suggestive of lower NR rate in HR+HER2− breast cancers accepting NACT

**Figure 3:**
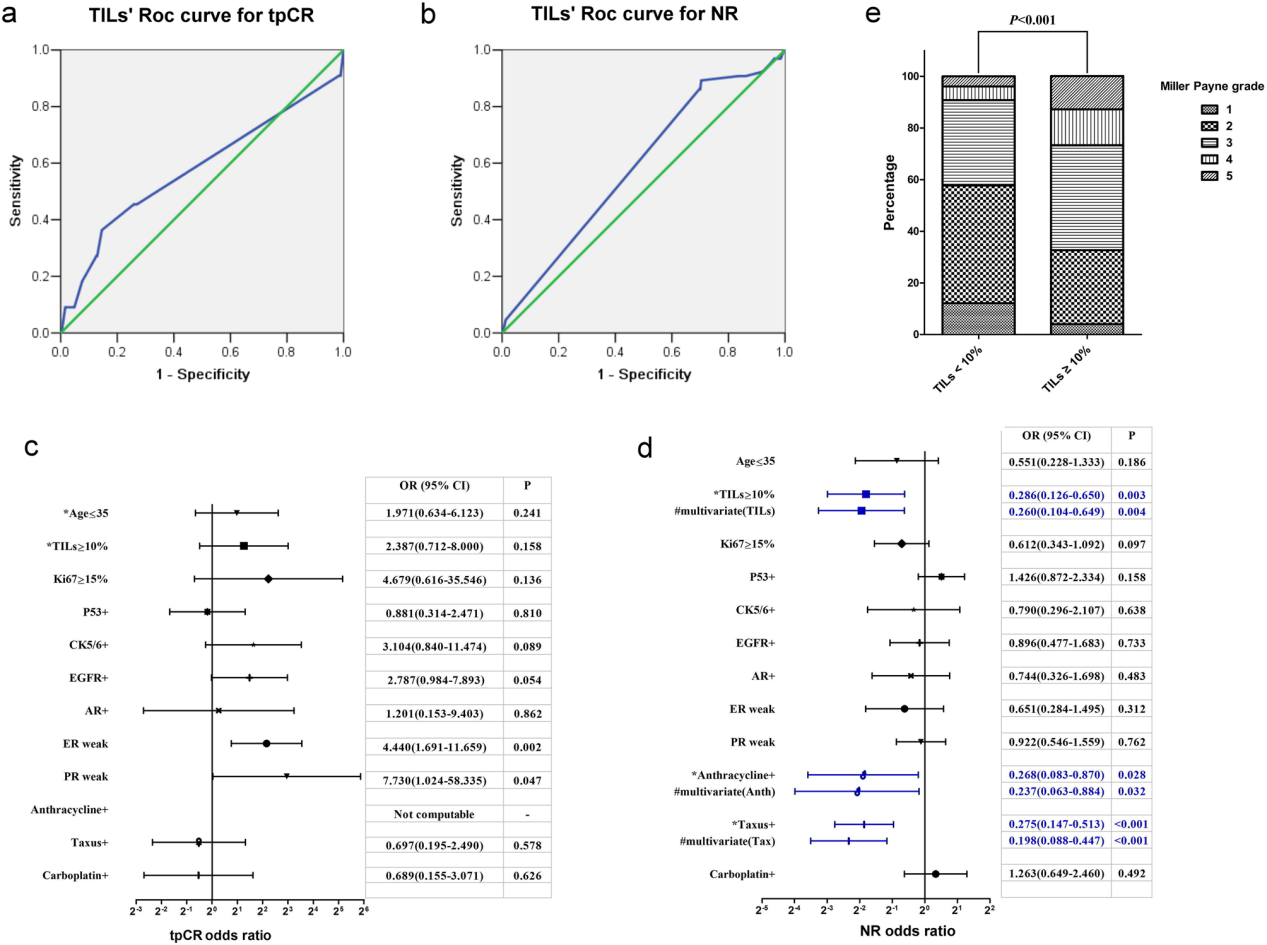
Analysis of factors including TILs on tpCR rate, NR rate, and Miller–Payne (MP) grading of HR+HER2− breast cancer cases after NACT. (**a**) ROC curve analysis of TILs on tpCR rate (AUC = 0.578, SE = 0.104, 95% CI = 0.375–0.782, P = 0.377). (**b**) ROC curve analysis of TILs on NR rate (AUC = 0.589, SE = 0.038, 95% CI = 0.516–0.663, P = 0.023). (**c**) Univariate and multivariate analyses of factors on tpCR rate. (**d**) Univariate and multivariate analyses of factors on NR rate. (**e**) The difference of MP grade between the high and low TILs groups was analyzed by nonparametric Mann–Whitney U test and P < 0.05 means statistically significant.

Of the 473 HR+HER2− breast cancer biopsy specimens, TILs were evaluated in 382 cases. The tpCR rate was 4.2%. ROC curve analysis suggested that TILs did not show significant predictive value for tpCR (AUC = 0.578, SE = 0.104, 95% CI = 0.375–0.782, P = 0.377, Fig. 3a). Univariate analysis of the tpCR was shown in Fig. 3c. And multivariate analysis failed to identify TILs as an independent factor to predict the tpCR response of cancer to NACT (Fig. 3c).

On the other side, the NR rate (82/473; 17.3%) was relatively high. ROC curve analysis confirmed that TILs showed significant predictive value for NR of HR+HER2− (AUC = 0.589, SE = 0.038, 95% CI = 0.516–0.663, P = 0.023, Fig. 3b). When TILs at a cutoff of 10, the Youden index reaches the maximum. Additional 166 consecutive HR+HER2− breast cancer cases receiving neoadjuvant chemotherapy as the validation set, their ROC curve and Youden index confirmed the rationality of 10% as the cutoff value for NR (Fig. S1a). Univariate analysis of the NR rate was shown in Fig. 3d. High TILs (TILs ≥ 10%) were associated with a lower NR rate {OR (95% CI): 0.285 (0.126–0.650); P = 0.003}. In the above validation dataset, univariate analysis of TILs ≥ 10% on NR also obtains similar statistically significant results. Multivariate analysis showed that high TILs {OR (95% CI): 0.260 (0.104–0.649); P = 0.004}, along with the administration of anthracyclines and taxanes were independent predictor for lower NR (Fig. 3b).

Of the 382 HR+HER2− cases with TILs’ information, 101 had high TILs and 281 had low TILs. The MP grading of the high TILs group was significantly higher than in the low TILs group (Z = 5.051; P < 0.001, Fig. 3e).

Using the non-parametric Spearman’s rank correlation coefficient test, it was found that positively correlation of TILs was identified with Ki67 index (R = 0.285, P < 0.001) and with MP grade (R = 0.250, P < 0.001), and negatively correlated was noted with ER (R = − 0.144, P = 0.005) and PR H-scores (R = − 0.107, P = 0.037, Table 2).

**Table 2:** Correlation of TILs with other pathological features in HR+HER2− cases receiving neoadjuvant chemotherapy.

Indicators	Cases	M(Q_R_)*	TILs	
			*r*_*s*_	*P*
Age	382	49(15.25)	− 0.030	0.560
ER (H-score)	382	180(130)	− 0.144	0.005
PR (H-score)	382	30(119)	− 0.107	0.037
Ki67 index (%)	363	30(30)	0.285	< 0.001
P53	379	1(20)	0.169	0.001
CK5/6	378	0(0)	0.135	0.009
EGFR	377	0(0)	0.164	0.001
AR (H-score)	376	120(120)	− 0.112	0.030
Miller Payne grade	382	–	0.250	< 0.001
Grade 1	38	–		
Grade 2	157	–		
Grade 3	134	–		
Grade 4	29	–		
Grade 5	24	–		

M(QR)*: Expressed by median (interquartile range).

### High TILs associated with a higher tpCR rate in HER2+ breast cancers accepting NACT combined with trastuzumab, and with a lower NR rate in those received only NACT

**Figure 4:**
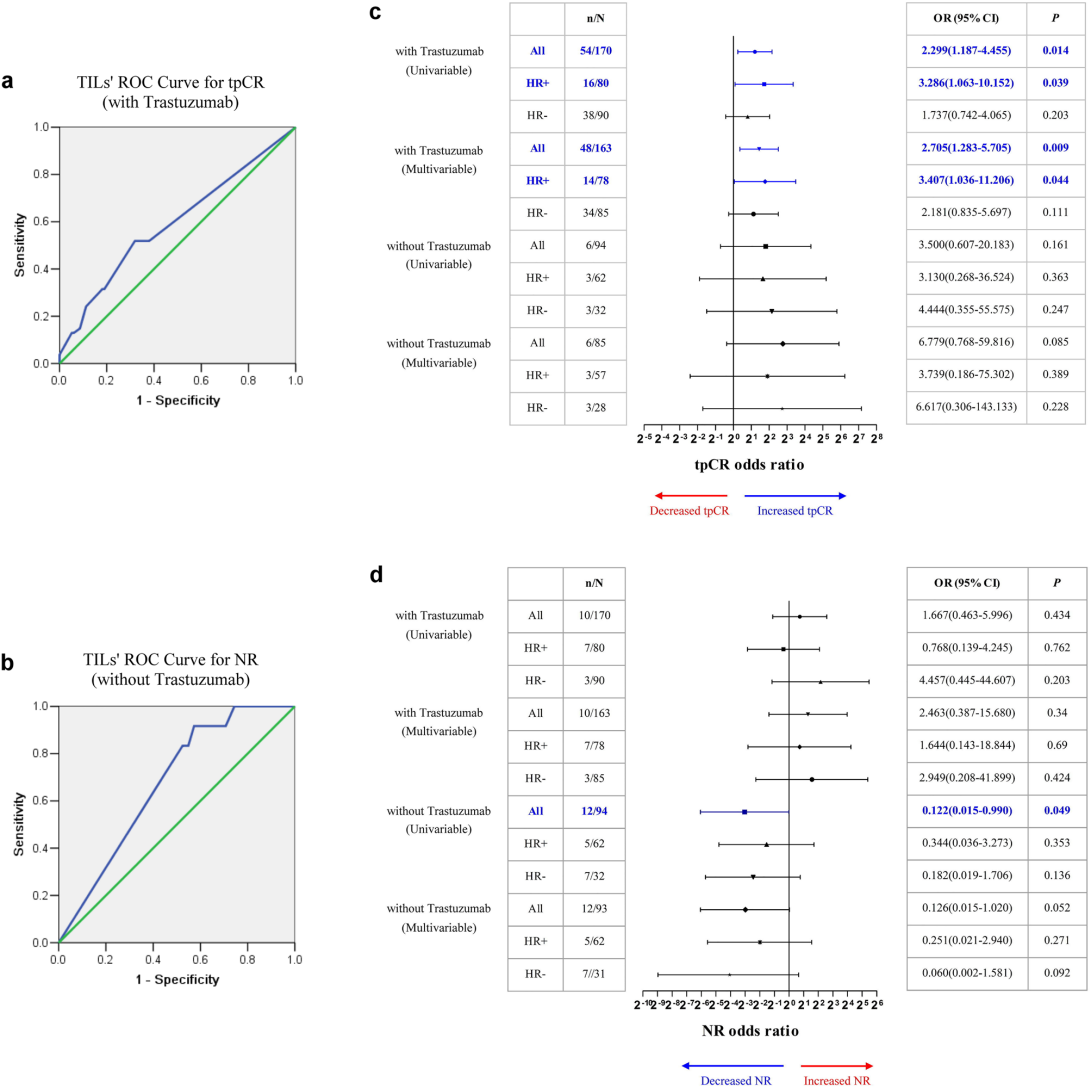
Analysis of TILs on tpCR rate and NR rate of HER2+ breast cancer after NACT combined with anti HER2 therapy or after NACT alone. (**a**) ROC curve analysis of TILs on tpCR rate (AUC = 0.592, SE = 0.048, 95% CI = 0.498–0.687, P = 0.053) of cases with anti HER2 therapy. (**b**) ROC curve analysis of TILs on NR rate (AUC = 0.674, SE = 0.069, 95% CI = 0.540–0.809, P = 0.052) with NACT alone. (**c**) Univariate and multivariate analyses of TILs on tpCR rate. (**d**) Univariate and multivariate analyses of TILs on NR rate.

Two hundred and twenty-three out of 354 (63.0%) patients with HER2+ cancer received NACT combined with anti-HER2 therapy, which included at least trastuzumab. ROC curve analysis suggested that TILs closed to have a predictive value of for their tpCR rate (AUC = 0.592, SE = 0.048, 95% CI = 0.498–0.687, P = 0.053, Fig. 4a). When TILs at a cutoff of 10%, the Youden index reaches the maximum. Additional 120 consecutive HER2+ breast cancer cases receiving neoadjuvant chemotherapy combined with trastuzumab as the validation set, their ROC curve and Youden index confirmed the rationality of 10% as the cutoff value for tpCR (Fig. S1b). In this subgroup, TILs’ status was assessed in 170 cases, among which 80 were HR+. Eighty cases (35.9%) achieved tpCR and 12 (5.4%) remained NR. Univariate analysis showed that tumors with high TILs (TILs ≥ 10%) had a significantly higher tpCR rate in all 170 cases {OR (95% CI): 2.299 (1.187–4.455); P = 0.014} and in 80 HR+ cases {OR (95% CI): 3.286 (1.063–10.152); P = 0.039, Fig. 4a}. Multivariate analysis also showed that high TILs was an independent predictor for high tpCR rate in all cases {OR (95% CI): 2.705 (1.283–5.705); P = 0.009}, and in HR+ cases thereof{OR (95% CI): 3.407 (1.036–11.206); P = 0.044, Fig. 4c}.

Remaining 131 patients with HER2+ breast cancer received only NACT. TILs’ status was assessed in 94 cases, among which 62 were HR+. Only 9 (6.9%) cases achieved tpCR but 16 (12.2%) remained NR. ROC curve analysis suggested that TILs closed to have a predictive value of for their NR rate (AUC = 0.674, SE = 0.069, 95% CI = 0.540–0.809, P = 0.052, Fig. 4b). When TILs at a cutoff of 10%, the Youden index reaches the maximum. Univariate analysis of TILs on tpCR rate and NR rate showed that high TILs (TILs ≥ 10%) correlated with a significantly lower NR rate in all 94 cases {OR (95% CI): 0.122 (0.015–0.990); P = 0.049, Fig. 4d}. Multivariate analysis showed a trend for high TILs to be an independent factor of low NR rate in all 94 cases {OR (95% CI): 0.126 (0.015–1.020); P = 0.052, Fig. 4d}.

Non-parametric Spearman’s rank correlation coefficient test revealed that in the 264 HER2+ patients with TILs’ assessment, no correlations of TILs with Ki67 index (R = 0.106, P = 0.092), ER (R = − 0.087, P = 0.159) or PR (R = -0.019, P = 0.756, Table 3) H-score.

**Table 3:** Correlation of TILs with other pathological features in HER2+ cases receiving neoadjuvant chemotherapy.

Indicators	Cases	M(Q_R_)*	TILs	
			*r*_*s*_	*P*
Age	264	51(15)	− 0.025	0.687
ER (H-score)	264	1(140)	− 0.087	0.159
PR (H-score)	264	0(10)	− 0.019	0.756
Ki67 index (%)	255	40(30)	0.106	0.092
P53	261	10(60)	0.084	0.176
CK5/6	258	0(0)	0.101	0.105
EGFR	258	0(30)	0.023	0.713
AR (H-score)	256	120(130)	0.033	0.595

M(Q_R_)*: Expressed by median (interquartile range).

### High TILs associated with lower NR rate in TNBCs accepting NACT

**Figure 5:**
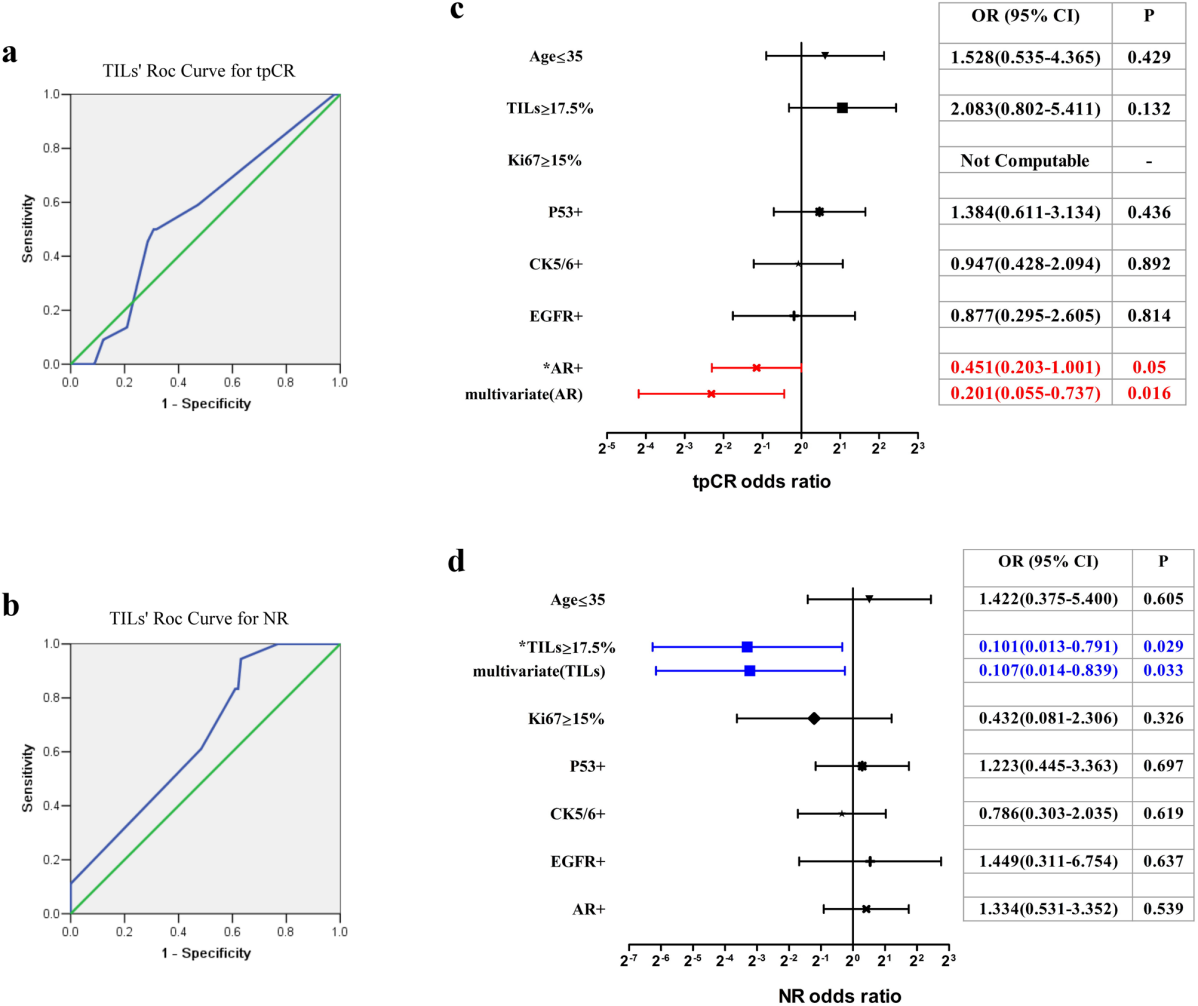
Analysis of factors including TILs on tpCR rate and NR rate of TNBC cases after NACT. (**a**) ROC curve analysis of TILs on tpCR rate (AUC = 0.558, SE = 0.065, 95% CI = 0.430–0.686, P = 0.398). (**b**) ROC curve analysis of TILs on NR rate (AUC = 0.649, SE = 0.063, 95% CI = 0.525–0.773, P = 0.046). (**c**) Univariate and multivariate analyses of factors on tpCR rate. (**d**) Univariate and multivariate analyses of factors on NR rate.

Of the 164 TNBC biopsy specimens, TILs were assessed in 113 cases. Of these, 42 (25.6%) cases achieved tpCR and 21 (12.8%) remained NR. Univariate and multivariate analyses showed that TILs ≥ 10% was not an independent factor affecting tpCR rate and NR rate (data not shown). However, ROC curve analysis confirmed that TILs showed significant predictive value for NR (AUC = 0.649, SE = 0.063, 95% CI = 0.525–0.773, P = 0.046, Fig. 5b), but not for tpCR (AUC = 0.558, SE = 0.065, 95% CI = 0.430–0.686, P = 0.398, Fig. 5a). When TILs at a cutoff of 17.5%, the Youden index reaches the maximum for predicting NR. Additional 38 consecutive TNBC receiving neoadjuvant chemotherapy as the validation set, their ROC curve and Youden index confirmed the rationality of 17.5% as the cutoff value for NR (Fig. S1c). So, after adjusting the threshold to TILs ≥ 17.5%, 36 cases had high TILs and 77 cases had low TILs. Univariate analysis {OR (95% CI): 0.101 (0.013–0.791); P = 0.029} and multivariate analysis {OR (95% CI): 0.107 (0.014–0.839); P = 0.033, Fig. 5d} of NR rate showed that TILs ≥ 17.5% became the independent predictor for low NR rate. Additionally, only 1 of the 36 cases with TILs ≥ 17.5% showed NR. Univariate analysis {OR (95% CI): 0.451 (0.203–1.001); P = 0.050} and multivariate analysis {OR (95% CI): 0.451 (0.055–0.737); P = 0.016, Fig. 5c} of tpCR rate showed that AR positivity was the only independent predictor for low tpCR rate. Non-parametric Spearman’s rank correlation coefficient test revealed that in TNBC patients, TILs were not correlated with Ki67 index (R = 0.068, P = 0.477, Table 4), but demonstrated a correlation trend with MP grade (R = 0.177, P = 0.060, Table 4).

**Table 4:** Correlation of TILs with other pathological features in TNBC cases receiving neoadjuvant chemotherapy.

Indicators	Cases	M(Q_R_)*	TILs	
			*r*_*s*_	*P*
age	113	48(20)	0.111	0.241
Ki67 index (%)	112	70(30)	0.068	0.477
P53	112	40(70)	0.129	0.174
CK5/6	110	5(60)	− 0.161	0.094
EGFR	112	70(52.5)	0.029	0.763
AR (H-score)	111	0(60)	0.006	0.954
Miller Payne grade	113	–	0.177	0.060
Grade 1	12	–		
Grade 2	37	–		
Grade 3	24	–		
Grade 4	11	–		
Grade 5	29	–		

M(Q_R_)*: Expressed by median (interquartile range).

## Discussion

In this study, we defined NR as no significant response to NAT in primary or local lymph node metastases or both, confirmed by pathology evaluation. Since the earliest case was less than four years ago, there is not enough follow-up time to assess the prognosis. NR does not necessarily indicate a worse prognosis, especially in HR+HER2− breast cancer. However, cancers with NR have a relatively high risk of continual progression during NAT and may not guarantee an opportunity for radical excision after NAT.

Our study found that TILs were negatively correlated with the NR rate of HR+HER2− cancer in neoadjuvant setting, and multivariate analysis indicated TILs ≥ 10% in pre-neoadjuvant biopsy was proven to be an independent predictor for lower NR rate in this subgroup. As this group is the largest population of breast cancer and many of them may not response well to NACT regimens. The tpCR rate in this group is relatively low and tpCR rate is not clearly correlated with patient prognosis. Therefore, the main purpose of NACT is to reduce the stage of breast cancer for complete radical excision and conserving surgery. Hence, NACT should be given priority in patients with higher TILs levels under other equal conditions. Some experts do recommend early surgery for patients who have progressive disease (PD) after 2–4 cycles of NAT and are still operable^14^. Our results bring up a hope that the status of tumor TILs might guide oncologists in early selection of a most effective treatment plan for patients and avoid unnecessary negative treatment effects.

A similar association of TILs with neoadjuvant tumor effect was also identified in TNBC, although the significant cutoff was at a higher level of TILs ≥ 17.5% (Optimized by ROC curve). Multivariate analysis in our study showed that TILs at this level were independent predictor for lower NR rate. In our cohort, only 1 of the 36 TNBC with TILs ≥ 17.5% had NR, and TILs in TNBC showed a trend in correlating with MP grade. This indicates that NACT could be the first choice when TILs are ≥ 17.5% with compatible other conditions. Similarly, when TNBC patients with SD after 2–4 rounds of NACT, the regimen should be adjusted and the new chemotherapy plan could be implemented when TILs ≥ 17.5%. In addition, TILs is also proved to be strongly correlated with PD-L1 status. Indeed, it has been shown that TNBC patients exhibiting high TILs at level > 20% was almost entirely PD-L1+ by the Ventana SP142 immunohistochemical assay^23^. In this regard, evaluation of TILs in TNBC may also help the PD-L1 evaluation and immunotherapy selection^24^. Our data failed to prove that TILs ≥ 17.5% to be an independent predictor for higher tpCR rate in TNBC, which is different from what reported by Denkert et al.^22^. The TILs status (only 5.3% TILs ≥ 60%) and tpCR rate (25.6%) in our cohort were significantly lower than that in their study (30.1% TILs ≥ 60% and tpCR rate 36.8%).

Prat et al.^25^ study showed that compared with HR-HER2+ breast cancer, the tpCR rate obtained by NACT combined with trastuzumab was relatively low in HR+HER2+ ones (35.6% versus 19%). In our study, the tpCR rate of HR-HER2+ was as high as 42.2% and that of HR+HER2+ was only 20%. The multivariate analysis of our study showed that high TILs were an independent predictor for high tpCR in HR+HER2+ patients received NACT combined with anti HER2 therapy, while it was not found in HR−HER2+ patients. The reason why high TILs levels did not become an independent factor for high tpCR in HR−HER2+ patients may be that the majority of cases (77/90) did not receive dual targeted anti HER2 therapy with trastuzumab and pertuzumab. So TILs is expected to help us screen out the expected beneficiaries for HR+HER2+ cases with NACT combined with trastuzumab.

Our study has limitations. In our cohort, 131 of the HER2+ breast cancer patients did not receive anti-HER2 therapy in the neoadjuvant setting due to non-medical reasons, and its impact on this study is complicated. At one hand, these HER2+ breast cancer patients failed to receive anti-HER2 therapy, which could have made the tpCR rate in entire cohort underestimated. On the other hand, we found in this group of patients, high TILs was close to be an independent predictor for a lower NR rate, similar to those in TNBC and in HR+HER2−.

In conclusion, this study found that TILs’ status of breast cancer could predict the NR of tumors. The predictive value may facilitate triage of patients with HR+HER2− cancer or TNBC who may benefit from NACT. Likewise, HR+HER2+ cancer with high TILs treated with NACT plus trastuzumab may likely achieve tpCR. HR+HER2− breast cancer with low levels of TILs should be carefully treated with neoadjuvant chemotherapy, and other alternatives such as neoadjuvant endocrine therapy can be considered.

## Materials and methods

### Ethics statement

Human breast tissues were collected with written consent from patients prior to their participation in the study. The protocols for collection and analysis of the samples were approved by the Institutional Review Board of Tianjin Medical University Cancer Institute and Hospital, in accordance with the current revision of the Declaration of Helsinki.

### Human breast cancer specimens

**Figure 6:**
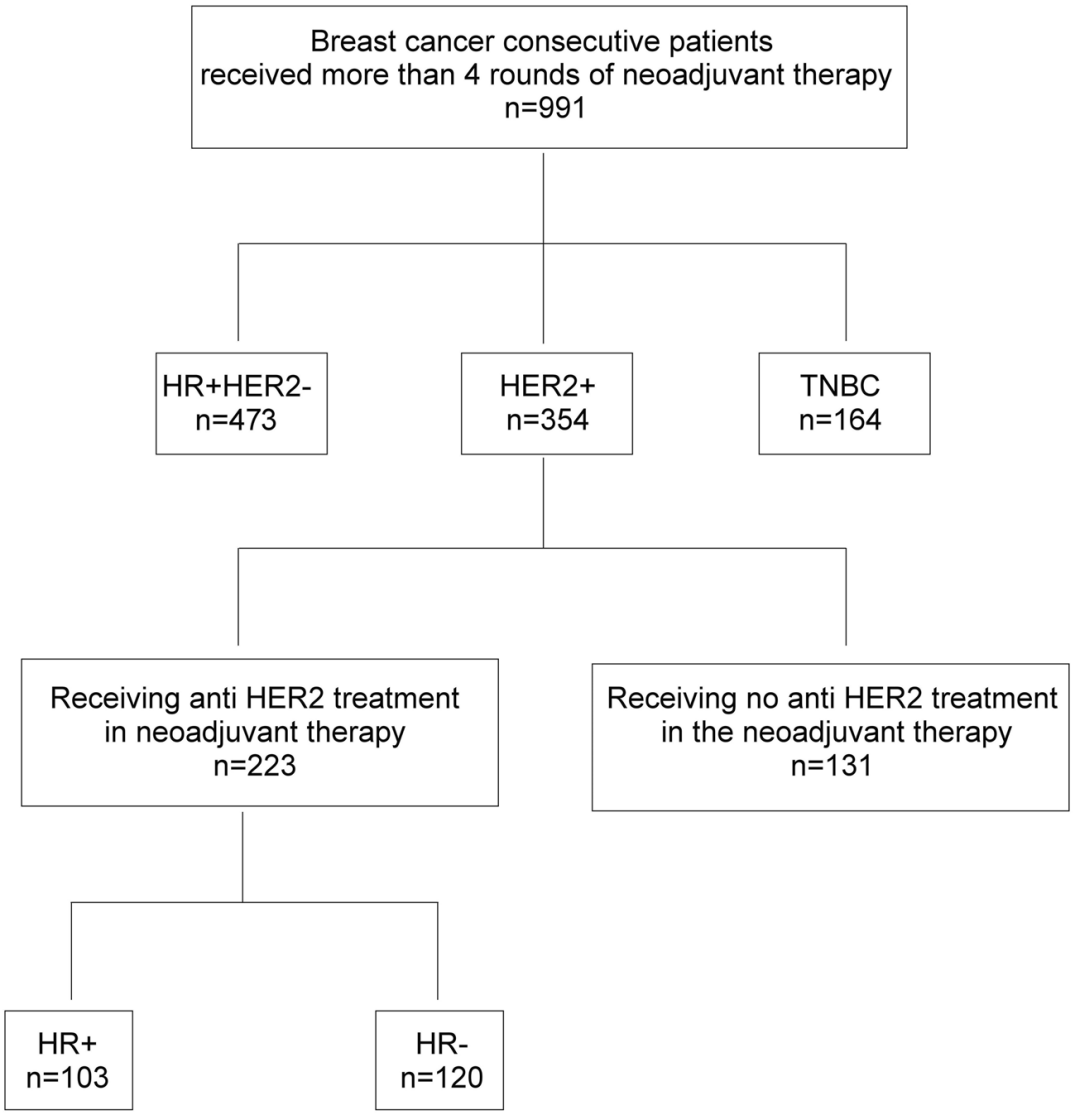
Study cohort. Needle biopsy specimens of 991 random cases that received NACT before breast conserving or radical mastectomy were included in this analysis. *HR* hormone receptor, including estrogen receptor α (ER), and progesterone receptor (PR), *TNBC* triple-negative breast cancer, *NACT* neoadjuvant chemotherapy.

Nine hundred ninety-one cases of invasive breast carcinoma excised after NACT were selected from a total of 6447 cases (15.4%) of excisional breast cancer samples in our hospital between October 1, 2018, and September 30, 2019. Among them, 473 cases were HR+HER2−, 354 cases were HER2+ and HR arbitrary (HER2+), and 164 cases were TNBC (Fig. 6). Each patient received at least 4 cycles of NACT. Two hundred and twenty-three HER2+ patients received standard NACT plus anti-HER2 neoadjuvant treatment. Pre-neoadjuvant biopsy cancer materials were used for the evaluations of pathological index including TILs. Among the 473 HR+HER− breast cancer cases, 51 cases were treated with anthracyclines only, 315 cases with anthracyclines + taxanes, and 54 cases with anthracyclines + taxanes + carboplatin. Among the 354 HER+ cases, 223 received NACT combined with anti-HER2 therapy. among them, 110 received combined treatment with taxanes and carboplatin, 188 received combined treatment with anthracyclines and taxanes, and 40 cases with anthracyclines + taxanes + carboplatins.Among the 164 TNBC cases, 85 received combined treatment with anthracyclines and taxanes, 3 received combined treatment with taxanes and platinum, and 32 received anthracyclines + taxanes + carboplatin.

To verify the reliability of the cutoff values obtained through ROC curves and Youden index, we also include 324 cases (166 HR+HER2−, 120 HER2+ and 38 TNBC) who received neoadjuvant treatment in our hospital from September 2022 to December 2022 and have completed radical or breastconserving surgery as a validation set.

### Evaluation of TILs

TILs were evaluated in 759 cases and scoring using the 2014 international TILs working group standard^26^. Tumor tissue sections stained with hematoxylin and eosin (H&E) were independently evaluated by two pathologists under light microscope to score TILs’ percent. TILs ≥ 10% was adopted as invasive breast cancer with high TILs, except in TNBC where the bar was adjusted to ≥ 17.5% to be considered as high TILs. The remaining 232 cases were biopsied at outside institutions and tissue materials were not available for the assay. TILs can be divided into tumor intraepithelial infiltrating lymphocytes and tumor interstitial infiltrating lymphocytes. The infiltrating lymphocytes in the interstitial cells are distributed in the interstitial cells between cancer nests and do not contact with cancer cells, so they are easy to judge and evaluate and reproducible. Therefore, currently, the main evaluation method is to evaluate the infiltrating lymphocytes in the interstitial cells.

### Evaluation of ER, PR, and AR in tumor cells

Immunohistochemistry (IHC) for Estrogen receptor α(ER) was performed with the BenchMark XT Automated IHC/ISH slide staining system (VENTANA) using an anti ERα ready-to-use antibody (rabbit monoclonal antibody, clone number SP1, VENTANA). Staining of progesterone receptor (PR) and androgen receptor (AR) was performed according to the methods in our previous publication^27^. Nuclear positivity of ER, PR, and AR ≥ 1% was defined as ER+, PR+, and AR+, respectively^28^. The expression levels of them were semi-quantified using the H-score^29^ system. ER and PR final scores lower than 100 were defined as weak ER and PR respectively.

### Evaluation of HER2 status in invasive breast cancer

The HER2 status of patients with invasive breast cancer was evaluated by IHC and fluorescence in situ hybridization (FISH). HER2 is defined as positive when HER2 IHC is 3+, or HER2 IHC is 2+ but gene amplification is proven by HER2 FISH^30^.

IHC for HER2 was performed with the BenchMark XT Automated IHC/ISH slide staining system (VENTANA) using an anti HER2/neu antibody (clone number 4B5, VENTANA). PathVysion HER2 DNA probe kit (Abbott Diagnostics) were used for FISH test according to the protocol recommended by the manufacturer. Each case was scored independently by two pathologists according to the 2018 ASCO/CAP Guidelines^30^.

### Evaluation of ki67 index and P53, CK5/6, and EGFR status in invasive breast cancer

IHC for EGFR was performed with a BenchMark XT Automated IHC/ISH slide staining system (VENTANA) using an anti-EGFR ready-to-use antibody (rabbit monoclonal antibody, clone number 5b7, VENTANA). Staining of Ki67, P53, and CK5/6 was performed according to the methods in our previous publication^27^.

### The Miller–Payne (MP) grading system

The MP grading system was used to evaluate the pathological responses of primary breast cancers which were divided into 5 grades: MP Grade 1–5, according to standards in the literature^10^.

### Definition of tpCR and NR

tpCR is defined as the absence of residual invasive cancer in breast and axillary nodes following NAT. Specimens with any remaining tumor cells in breast, vessels/lymph nodes were precluded from tpCR^8^. NR to NAT was defined as cases without obvious chemotherapy response in the primary tumor (Miller–Payne Grade 1: No change or some alternation to individual malignant cells but no reduction in overall cellularity^10^) or in metastatic lymph nodes (Classified as grade N–C according to the Sataloff grading system for lymph nodes: Lymph nodes with cancer cell metastasis without chemotherapy response^11^).

### Statistical analyses

ROC curves were built using SPSS 15.0 for Windows. Mann–Whitney U tests were used to determine the significance of the differences between two groups of data with a skewed distribution. The correlations between two variables of non-normal distribution were analyzed using non-parametric Spearman’s rank correlation coefficient tests. Odds ratios (ORs) with a 95% confidence interval (95% CI) were calculated by logistic regression analysis. Age, TILs, Ki67 index, P53, CK5/6, EGFR, ER, PR, AR, and the use of Anthracycline, Taxus and Carboplatin were included in multivariate logistic analysis for tpCR and NR status. A *two-sided P* < 0.05 was considered statistically significant in all analyses.

## Supplementary Information

Supplementary Legends.

Supplementary Figure S1.

Supplementary Table S1.

Supplementary Table S2.

## Data Availability

All data generated or analysed during this study are included in this published article (and its Supplementary Information files).

## Abbreviations

TILs: Tumor-infiltrating lymphocytes
tpCR: Total pathological complete remission
NACT: Neoadjuvant chemotherapy
NR: Nonresponse to NACT
HR: Hormone receptor
TNBC: Triple-negative breast cancer
NAT: Neoadjuvant therapy
MP grade: Miller–Payne grade
OR: Odds ratio
ROC curve: Receiver operating characteristic curve
95% CI: 95% Confidence interval
AUC: Area under curve
SE: Standard error
M(Q_R_): Median (interquartile range)
