# Supplementary material for: Effects of tumor-infiltrating lymphocytes on nonresponse rate of neoadjuvant chemotherapy in patients with invasive breast cancer
Source: Sci Rep. 2023 Jun 7;13:9256. doi: 10.1038/s41598-023-36517-2 (PMC10247751; doi:10.1038/s41598-023-36517-2)
Supplement: Supplementary file 1 — Supplementary Legends. [file 41598_2023_36517_MOESM1_ESM.docx]

**Supplementary Information**

**Table S1** Raw data of the study cohort of neoadjuvant chemotherapy cases.

**Table S2** Raw data of the validation set of neoadjuvant chemotherapy cases.

**Figure S1 TILs’ predictive value and optimal cutoff on tpCR or NR rate in the validation set.** (a) TILs’ ROC curve for NR in 166 consecutive HR + HER2 - cases; (b) TILs’ ROC curve for tpCR in 120 consecutive HER2 + cases (All patients received neoadjuvant chemotherapy combined with trastuzumab); (c) TILs’ ROC curve for NR in 38 consecutive TNBC
