# Supplementary figures and images for: Effects of tumor-infiltrating lymphocytes on nonresponse rate of neoadjuvant chemotherapy in patients with invasive breast cancer
Source: Sci Rep. 2023 Jun 7;13:9256. doi: 10.1038/s41598-023-36517-2 (PMC10247751; doi:10.1038/s41598-023-36517-2)

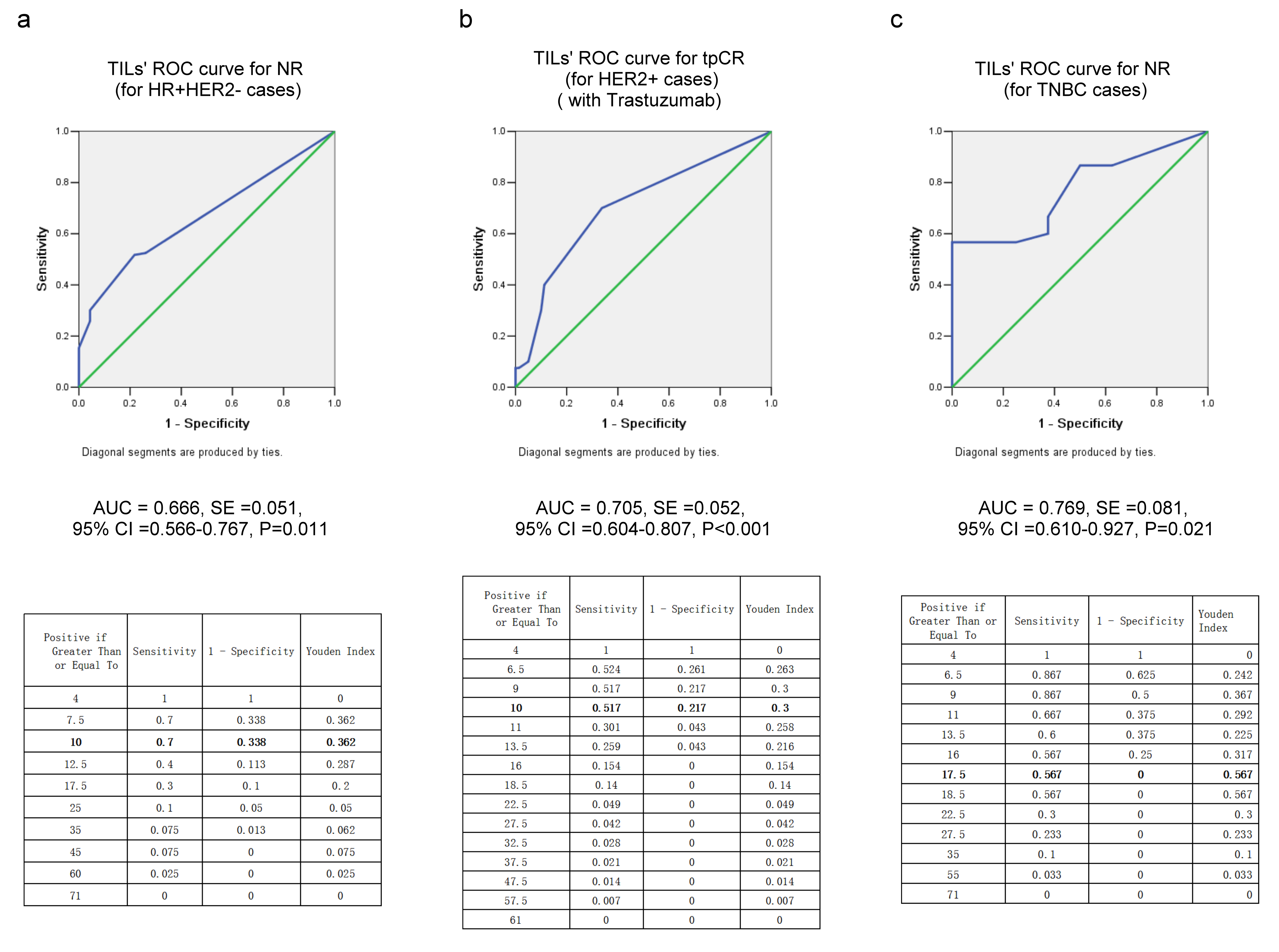

Supplement: Supplementary file 2 — Supplementary Figure S1. [file 41598_2023_36517_MOESM2_ESM.tif]
